# Raman amplification for trapped radiation in crystalline single Si nanoparticle

**DOI:** 10.1038/s41598-023-27839-2

**Published:** 2023-01-18

**Authors:** G. Mannino, M. Condorelli, G. Compagnini, G. Faraci

**Affiliations:** 1 CNR-IMM, Zona Industriale Strada VIII N°5, 95121 Catania, Italy; 2 Dipartimento di Scienze Chimiche, Università di Catania, Via Santa Sofia 64, 95123 Catania, Italy; 3 Dipartimento di Fisica e Astronomia, Università di Catania, Via Santa Sofia 64, 95123 Catania, Italy

**Keywords:** Materials science, Nanoscale materials

## Abstract

In a single crystalline Si particle, we observed a huge amplification of the Raman peak at 521 cm^−1^. With an AFM microscope, coupled with a Micro-Raman spectrometer, we investigate a single Si particle at wavelengths of 532 nm, 633 nm, and 785 nm. As observed by transmission electron microscopy, it has an octahedral shape of 150 nm in size. Thermal effects were detected on the Raman peak when the laser radiation, trapped inside, determines the heating of the particle up to its fusion. In these cases, the Raman peak splits into two components, the first at the crystal position and the other shifted at a lower value. The data permit the identification of the amplification mechanism of the Raman peak as trapped radiation moving forward and backwards into the particle. The thermal effects are attributed to phonon confinement and reduced thermal exchange with the surrounding. The present results are discussed in light of local order, the uncertainty principle, and phonon dispersion curves, and corroborated by shape-dependent simulation of absorption, scattering, and extinction behaviour.

## Introduction

The properties of nanoparticles, as amplifying systems, have been emphasized in many papers, regarding a large variety of materials, such as Si^1–13^, C^14^, Ge^15^, and others. We limit here to mention only Si nanostructures, showing amplification properties, observed by Raman spectroscopy, as discussed in several review papers^16–21^. Of course, any effort to determine the amplification mechanism of radiation absorption, scattering and/or emission, not only in the visible range, is valuable being related to energy production. Furthermore, for practical and economic reasons, the size range of around a hundred nanometres is particularly important because it avoids the difficulties related to quantum confinement and momentum enlargement^22^.

The principal fields of applications are the optical and electrical ones since they are strongly related to several devices. Giant photoluminescence emission^2,23–25^, surface-enhanced stimulated emissions^26^, whispering-gallery sensors^27^, and carrier multiplication in photovoltaics^28^ are of particular interest for the involved applications.

A huge enhancement of the first-order Raman peak was recently reported for Si nanoparticles (Si-NP)^25^. These Si-NP also showed an enormous photoluminescence emission, with nonlinear thermal behaviour. In these cases, the sample was made of agglomerated Si-NPs forming a porous layer up to a few microns in thickness. Two concurrent mechanisms were suggested to justify the large Raman peak amplification and the enhancement of radiative production^25,29^. The first was ascribed to the single-photon contribution whenever the radiation is trapped and scattered several times inside the Si-NP before absorption or escape. The second depends on the cooperative scattering effects of the photons on the Si-NPs whenever they are very close to each other.

The possibility of Raman phenomena resonant with electron transitions induced by the laser beam radiation, larger than the Si energy gap, was also considered. However, no such effect was detected at any of the laser energies used, confirming the absence of resonant phenomena in the explored wavelength range.

Here, we investigate a single crystalline Si-NP on a graphite substrate to discriminate precisely whether the first mechanism alone is sufficient to enhance the Raman response, distinguishing the relative contributions.

## Results and discussion

**Figure 1:**
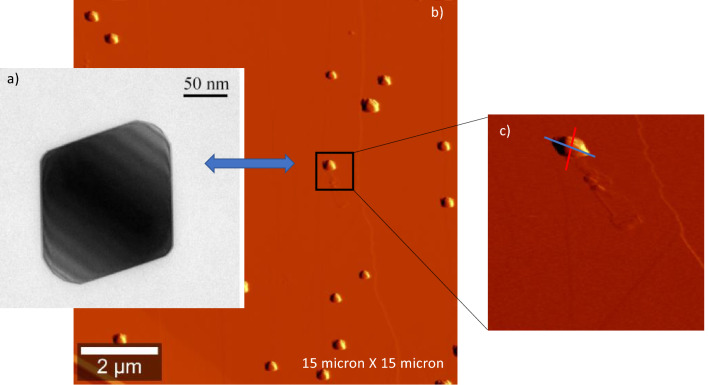
(**a**) Transmission electron microscopy image of a crystalline Si-NP; (**b**) Atomic force microscopy image of single Si-NP deposited on a HOPG substrate; (**c**) the enlarged area represents the area under the Raman laser beam.

In Fig. 1a we show a transmission electron microscopy (TEM) image of a crystalline Si-NP. Using an apparatus where a micro-Raman spectrometer is coupled with an atomic force microscope (AFM), we have been able to locate a single Si-NP under the laser beam of the Raman detector. The apparatus is equipped with three lasers having wavelengths of 532 nm (maximum power 14mW), 633 nm (maximum power 10mW), or 785 nm (maximum power 25mW). The spot of the laser of about 1 µm is larger than the size of a Si-NP as indicated in the inset of Fig. 1b,c where the AFM image of Si-NPs on a HOPG substrate is shown. The laser spot radius, certainly larger than the Si-NP footprint, is also much smaller than the interparticle distance.

**Figure 2:**
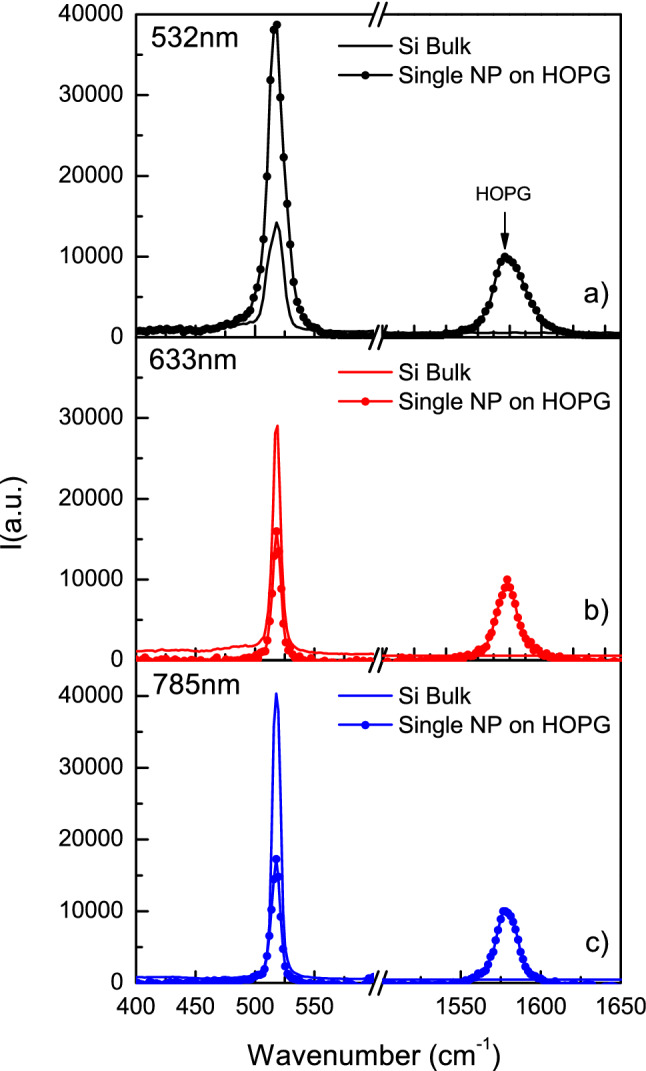
Raman spectra (acquisition 100 s) at three laser wavelengths 532 nm (**a**), 633 nm (**b**), and 785 nm (**c**) of a single Si-NP deposited on a HOPG substrate. All spectra have been normalized to the HOPG peak. Bulk Si spectra are collected in the same laser power conditions.

In Fig. 2a Raman spectra are reported for a Si-NP with the size of 150 nm under various laser radiation wavelengths each with a laser power of 20% of the maximum at that wavelength.

For convenience, the spectra have been normalized to the peak height of the signal coming from the graphite substrate while a reference spectrum is taken on a Si crystal wafer at the same laser power. It’s noteworthy that the raw spectrum area of a single Si-NP is higher than that of a 500 µm thick bulk Si wafer almost by a factor of 4 in the case of 532 nm. However, this amplification is even higher when considering the volume probed in the Si-NP (1.9 × 10^–15^ cm^3^) compared with the volume probed in the Si wafer that, for a laser spot (1 µm^2^) multiplied by the 532 nm light penetration depth of 1.3 µm, is of 4 × 10^–12^ cm^3^. Therefore, the volume-corrected amplification factor is A_532nm_ = 6700. Similarly, in Fig. 2b at 633 nm wavelength (Si bulk probed volume of 9.6 × 10^–12^ cm^3^), the amplification is A_633nm_ = 2500, and in Fig. 2c at 785 nm wavelength (Si bulk probed volume of 3.4 × 10^–11^ cm^3^), the amplification is A_785nm_ = 7500.

Such intense signal amplification is attributed to the longer travelling path of the radiation inside the Si-NP, due to multiple reflections before absorption and escape. Other possible amplification mechanisms such as a cooperative scattering of radiation escaping the Si-NP, the possibility of near Si-NP acting as Raman scattering enhancers^2^ or whispering gallery mode^27^ are excluded for a single Si-NP. Furthermore, we observe that no shift is detected in the Raman spectra of Si-NP. This implies that, whenever we maintain the power of the laser low enough (~ 20% of the maximum power, see Fig. 2) for a given duration of the irradiation (100 s), we avoid any phonon increment and therefore the temperature does not increase. In fact, in Fig. 2, no shift of the Raman peak is visible, denoting a complete dispersion of the heat produced by the laser beam.

**Figure 3:**
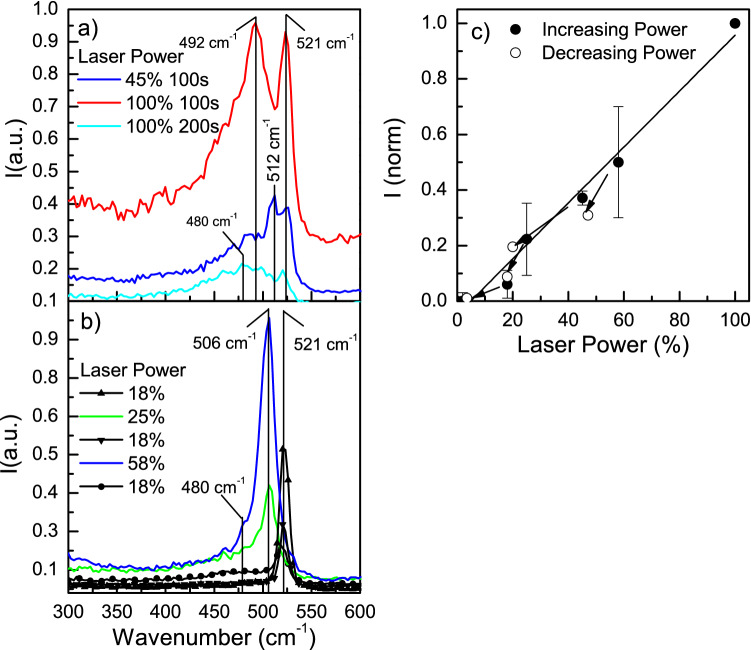
(**a**) Raman spectra were taken at 532 nm for different laser power as specified in the figure (% of the maximum power). At the maximum power (100%) a 100 s irradiation time produces a partial melting of the Si-NP while a 200 s irradiation time a full melt; (**b**) Raman peak intensity of the crystal component as a function of the laser power, showing, during a sequence of heating/cooling processes the partial melting/crystallization process of the Si-NP. (**c**) Normalized Raman peak intensity of the 521 cm^−1^ crystal component as a function of the laser power, showing a linear dependence of the peak position on the irradiation power;

On the contrary, if the laser power is increased to 45% of its maximum power, a splitting of the Raman peak is observed (Fig. 3a), with a component still present at 521 cm^−1^ but another component at a lower wavenumber (512 cm^−1^). With increasing the laser power to its maximum (i. e. increasing the temperature inside the Si-NP) this component moves to lower wavenumbers from the crystal position down to 492 cm^−1^ denoting a significant warming up of the Si-NP. This “warm” component is attributed to an increase in local temperature caused by the reduced thermal exchange. It is reasonable to suppose that not the entire particle is at the same temperature (in that case one would expect one single peak shifted) but that the internal part of the Si-NP stands at a higher temperature than the surface part, just because the exterior of the Si-NP has better heat exchange with the substrate and the surroundings. This implies a cold and a warm portion, both still crystalline. This coexistence produces two Raman peaks of which one is shifted to lower wave vectors^4^.

The higher temperature determines phonon increment and bond expansion. The higher the temperature, the longer the bonds are, up to the breaking of long-range crystalline order. If the maximum laser power is held briefly (100 s) the Si-NP has still a crystalline fraction (see Fig. 3a). Doubling irradiation times to 200 s, the fusion of the Si-NP is reached, and, being absent any crystal part that can act as crystallization seed, a unique peak at 480 cm^−1^ of the amorphous state is detected due to the confluence of both structures after cooling. As visible in Fig. 3b (solid symbols) the intensity of the Raman signal related to the crystalline component follows a linear trend with the laser on a normalised power scale. The heating process is to a certain extent reversible as shown in Fig. 3b if a threshold (~ 60% of the maximum power) is not passed. If after cooling, a second process is performed on the same Si-NP (open symbols), the Raman peak is restored to its standard position at around 521 cm^−1^ and sits close to linearity. However, as evidenced in Fig. 3c, although the position follows a linear trend, the intensity of the Si peak is lower even if using the same laser power (18% of the maximum power). Here, all 5 irradiations at various powers and identical times (100 s) have been done on the same Si-NP in sequence, leaving time for the Si-NP to cool down after each irradiation. The reason why the intensity decreases is that very likely, the cooled crystal contains defects i.e., some part remains amorphous. This defective content increases and becomes well visible in the last irradiation at 18% (filled circle) where the amorphous component at 480 cm^−1^ is visible.

We discuss how the previous results remark on the novelty of the present data: we have been able to identify the amplification mechanism of Raman enhancement, just by investigating a single Si-NP on a graphite substrate and observing the Raman response. This does not exclude the other mechanism indicated when Si-NPs are agglomerated in thick layers but permits attributing the internal trapping contribution to the related amplification factor in the case of a single Si-NP. Furthermore, an interesting splitting of the Raman peak reveals a double mixed state due to an excitation of the Si–Si bonds caused by phonon confinement increment in the Si-NP. The consequent expansion of a bond can increase up to its breaking. This explains why the Si-NP can lose its long-range crystal state, maintaining only a short-range order Si–Si, i.e., becoming amorphous.

**Figure 4:**
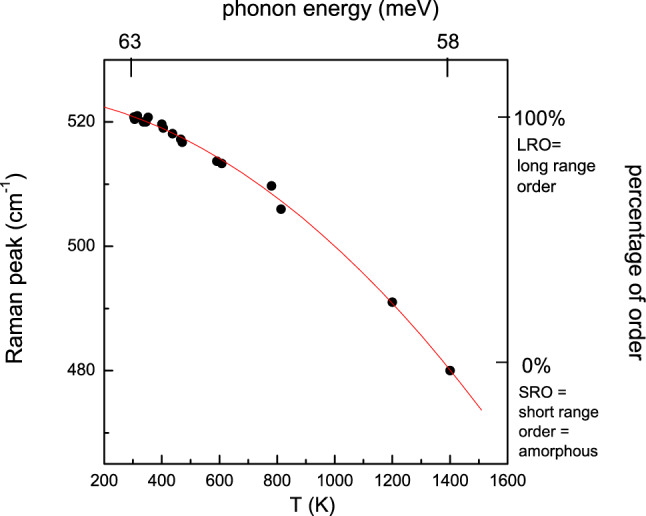
Plot of Raman peak as a function of the temperature, from the crystalline to the amorphous state. The phonon value at k = 0 is 63 meV, whereas it reduces to 58 meV for the amorphous configuration, where the values of k are averaged over the range permitted by the uncertainty principle. This is confirmed by the width at half maximum of the curves (not reported here) very wide for amorphous, whereas in crystals is very thin.

As well known, in Raman first-order scattering, the k ~ 0 phonon selection rule is valid only for crystalline states with long-range order. In other words, the phonons^29^ contributing to the first-order Raman scattering of a photon should have a negligible wave vector because of energy–momentum conservation rules. Therefore, phonons at k ~ 0, in the optical branches^29^, contribute to their high density of states in a crystal, because of long-range order. Otherwise, as soon as the crystal state begins to be lost because of bonds bending, as in amorphous states, and/or for partial bond thermal expansion, the uncertainty principle Δr Δk ~ 1 (r = interatomic distance, i.e., nearest neighbour bond with crystal order, k = wavevector), imposes a larger value of k around the 0 value^22^. This implies an average over the phonon optical dispersion curves, causing a reduced value of the phonon energy from the value 63 meV (k = 0), down to 58 meV in the amorphous state with a Raman peak at 480 cm^−1^. In Fig. 4 we show in fact how are related these quantities: Raman shift, temperature, phonon energy, and lattice order. Also, the broad FWHM (full width at half maximum) of the amorphous Raman peak reflects the large phonon contribution imposed by the Uncertainty Principle when the order in Si crystal lattice reduces only to nearest neighbours, as in amorphous states, because of random bonds bending. The phonon optical branches decrease from the top value (63 meV at k = 0) of about 8% along with the Brillouin zone symmetry directions and as a consequence, a weighted average over the values permitted by the uncertainty principle, taking into account the density of phonon states, should produce a broad Raman peak, as in fact detected for the amorphous silicon with an FWHM = 200 cm^−1^.

To corroborate the previous interpretation of the results, we simulated the optical response of a single Si-NP under the laser beam. We used commercial software that relies on the finite difference time domain method (FTDT) to solve Maxwell’s equation.

**Figure 5:**
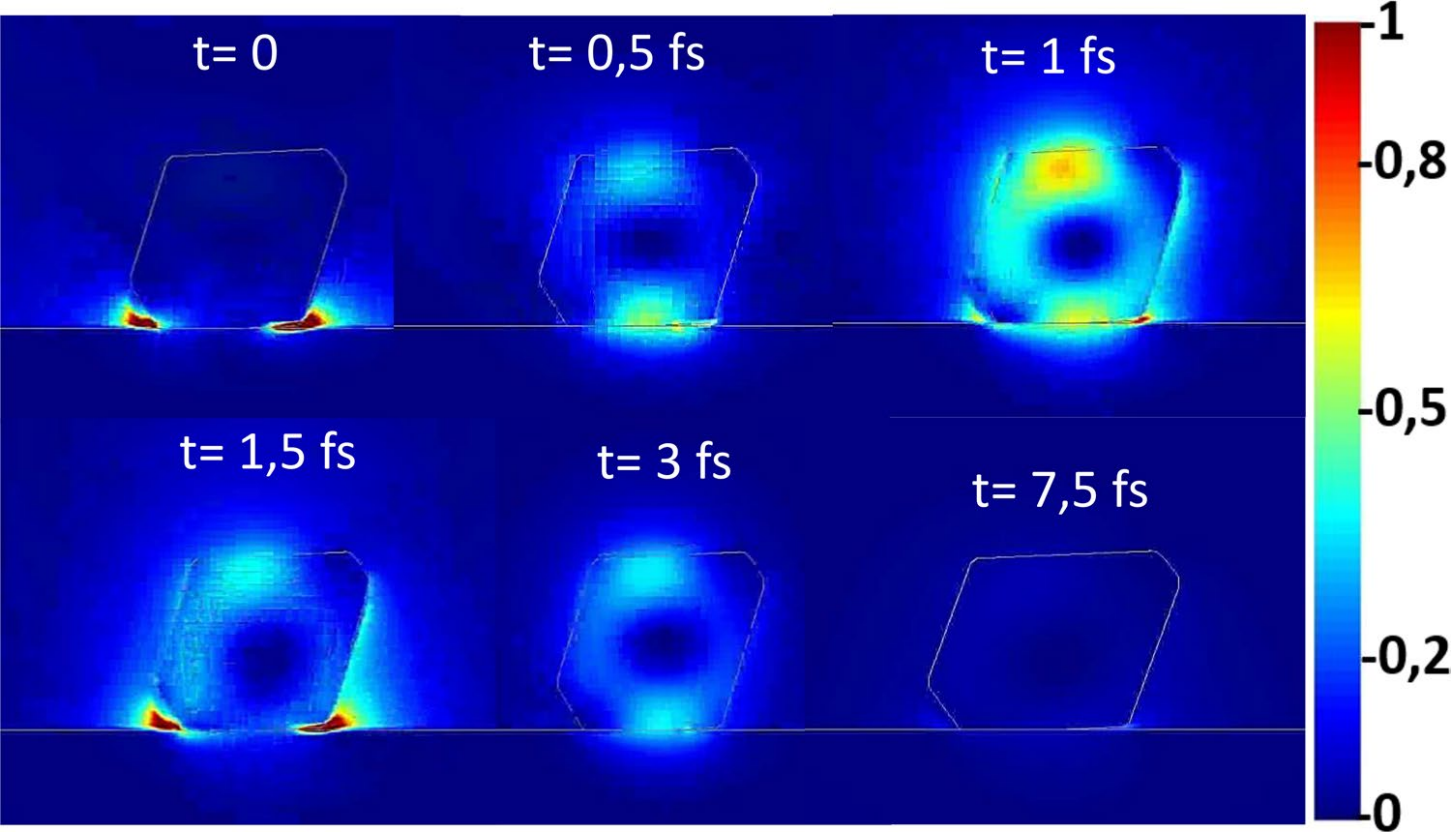
Normalised electric field intensity on colour scale inside the Si-NP exposing [111] facet after irradiation with 532 nm laser attesting the light trapping at the base of the Raman signal amplification.

The images reported in Fig. 5 represent the output intensity of the transmitted electric field of the modelled nanostructure at the wavelength used for the Raman study.

The Si-NP has been placed with the largest [111] face parallel to the sample surface being the most likely configuration experimentally observed here (see Fig. 1) and in Ref 24. The laser pulse of the signal lasts a few femtoseconds and t is set to 0 when the light has all passed through the sample, being for all three wavelengths the absorption length much greater (1.3 µm, 3 µm and 10 µm) than the Si-NP size (150 nm). Subsequently, we show (Fig. 5) the frames relative to 0.5–1–1.5–3 and 7.5 femtoseconds following the source switch-off (t = 0), extracting the relative images which represent the intensity of the electric field E^2^(t) (V/m)^2^ in absolute value. The colour scale represents the electric field from minimum ((blue) to maximum (red) intensity.

The electromagnetic field propagates inside the nanostructure, for several fs, when the exciting light is turned-of due with an enhanced intensity thanks to scattering and reflection phenomena at the inside border of the nanostructure. Once again, this phenomenon and the relative intensity reach their maximum when the nanostructures are irradiated with a wavelength of 532 nm. This latter result confirms the first mechanism of propagation and reflection of the photon inside the nanostructure proposed in this paper. Moreover, the high intensity of the electric field in the proximity of the nanostructure surface, together with the mechanism of reflection and scattering of the photon inside it, explain the highest intensity of the phonon contribution and the related temperature increase. The full propagation behaviour of the electric field for all three wavelengths is shown in a video (see Supplementary materials Figs. S1, S2 and S3) where the complete electromagnetic field propagation is shown as a function of the simulation time.

## Conclusions

The previous results are of particular importance from an applicative point of view. Trapping of photons in a Si-NP with consequent radiative amplification also implies electron excitation enhancement from the valence to the conduction band since the photon energy largely exceeds the Si band gap. The evident electron concentration amplification in the conduction band could be used to increment a photovoltaic current in a solar energy conversion device^30,31^.

## Methods

### Nanoparticle synthesis

We used an inductively coupled plasma chemical vapour deposition equipment to obtain monocrystalline, single, monodisperse, and single-shaped Si-NP. Samples are prepared in vacuum (base pressure < 10^–6^ Torr) in a reactor with a process pressure of 20mTorr in a way that high-density plasma ensures the capability of this ‘‘non-thermal’’ plasma to enhance crystalline Si-NP formation rather than SiH_4_ decomposition into amorphous Si deposition. This reactor has a vertical geometry, the plasma is highly ionized (> 5 × 10^11^ ions/cm^3^), and the substrate is maintained at a low temperature (50 °C). The deposition is performed on a circular area with a diameter of 6 inches. The density of the Si-NP has been maintained as low as possible (< 1 × 10^8^/cm^2^) to avoid overlap. As a substrate, we used graphite to avoid any contribution to the Raman peak.

### Raman measurements

For the Raman analysis, a micro-Raman Witech alpha R with 3 lasers with 3 different wavelengths, 532 nm (maximum power 14mW), 633 nm (maximum power 10mW), and 785 nm (maximum power 25mW) were used. The analysis parameters used are always the same specifically, we acquired 10 spectra with 10 s of integration time (100 s) using a 100X objective. In one case (see Fig. 3a) the acquisition time has been 200 s. AFM measurements are performed with the same instrumentation in alternate contact mode with a resolution of 256 points for 256 lines.

### Light trapping simulation

Theoretical calculation of the enhanced electromagnetic field and its propagation inside the nanostructure, under illumination by monochromatic light (532, 633, 785 nm) were carried out with Lumerical FTDT by ANSYS^32^, simulation software that relies on the finite difference time domain method (FTDT) to solve Maxwell’s equation. The propagation vector of the exciting wavelength is perpidicullary direct to the up face of the nanostructure (Z axes) and is orthogonally polarized (Y Axes). The electric field for each wavelength is normalised to its and reported as a colour scale from minimum (blue) to maximum (red).

## Supplementary Information

Supplementary Information.

## Data Availability

The datasets used and/or analysed during the current study are available from the corresponding author upon reasonable request.
