# Supplementary material for: Raman amplification for trapped radiation in crystalline single Si nanoparticle
Source: Sci Rep. 2023 Jan 18;13:1014. doi: 10.1038/s41598-023-27839-2 (PMC9849211; doi:10.1038/s41598-023-27839-2)
Supplement: Supplementary file 1 — Supplementary Information. [file 41598_2023_27839_MOESM1_ESM.pptx]

## Slide 1
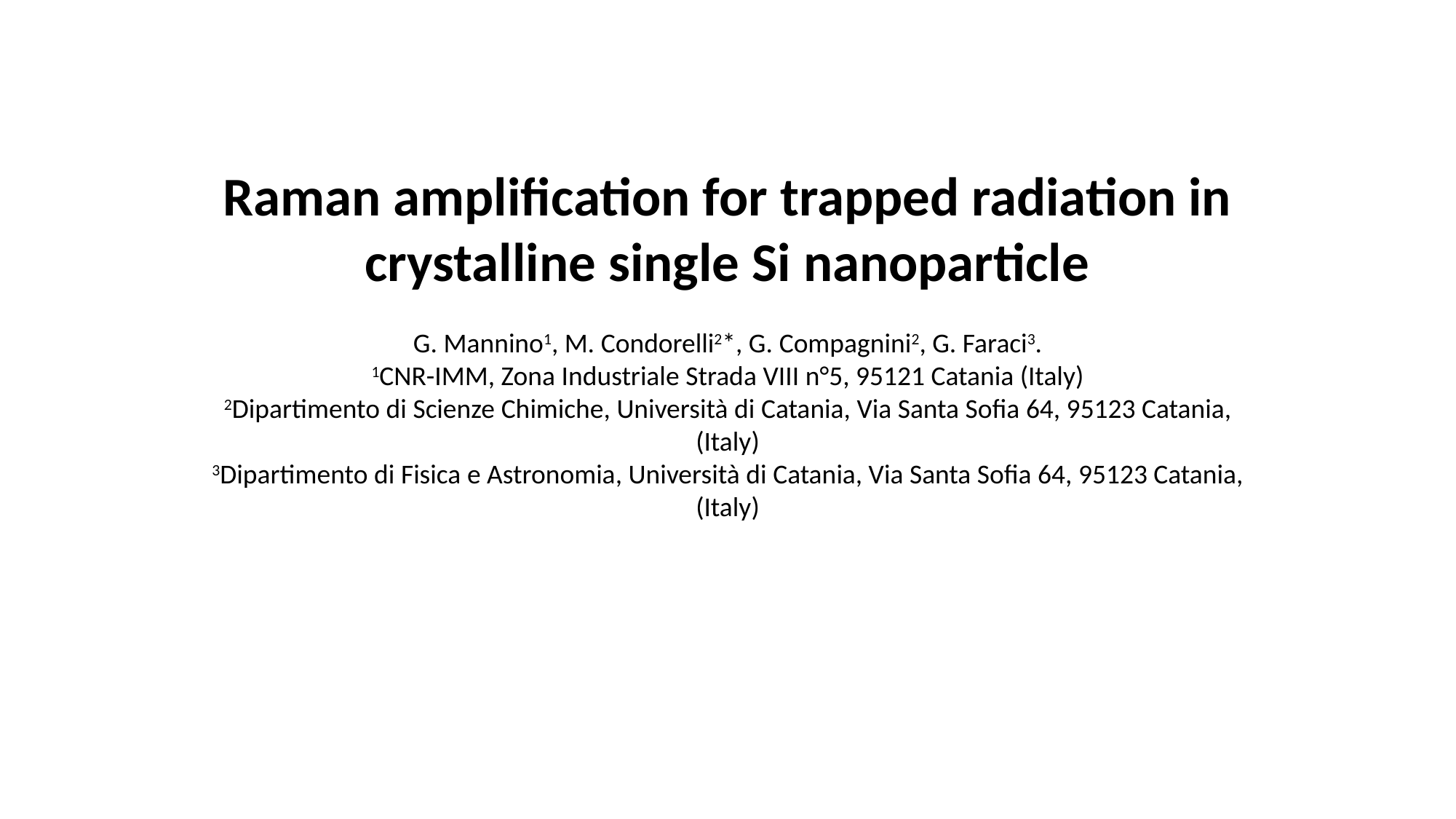

Raman amplification for trapped radiation in crystalline single Si nanoparticle
G. Mannino1, M. Condorelli2*, G. Compagnini2, G. Faraci3.
1CNR-IMM, Zona Industriale Strada VIII n°5, 95121 Catania (Italy)
2Dipartimento di Scienze Chimiche, Università di Catania, Via Santa Sofia 64, 95123 Catania, (Italy)
3Dipartimento di Fisica e Astronomia, Università di Catania, Via Santa Sofia 64, 95123 Catania, (Italy)

## Slide 2
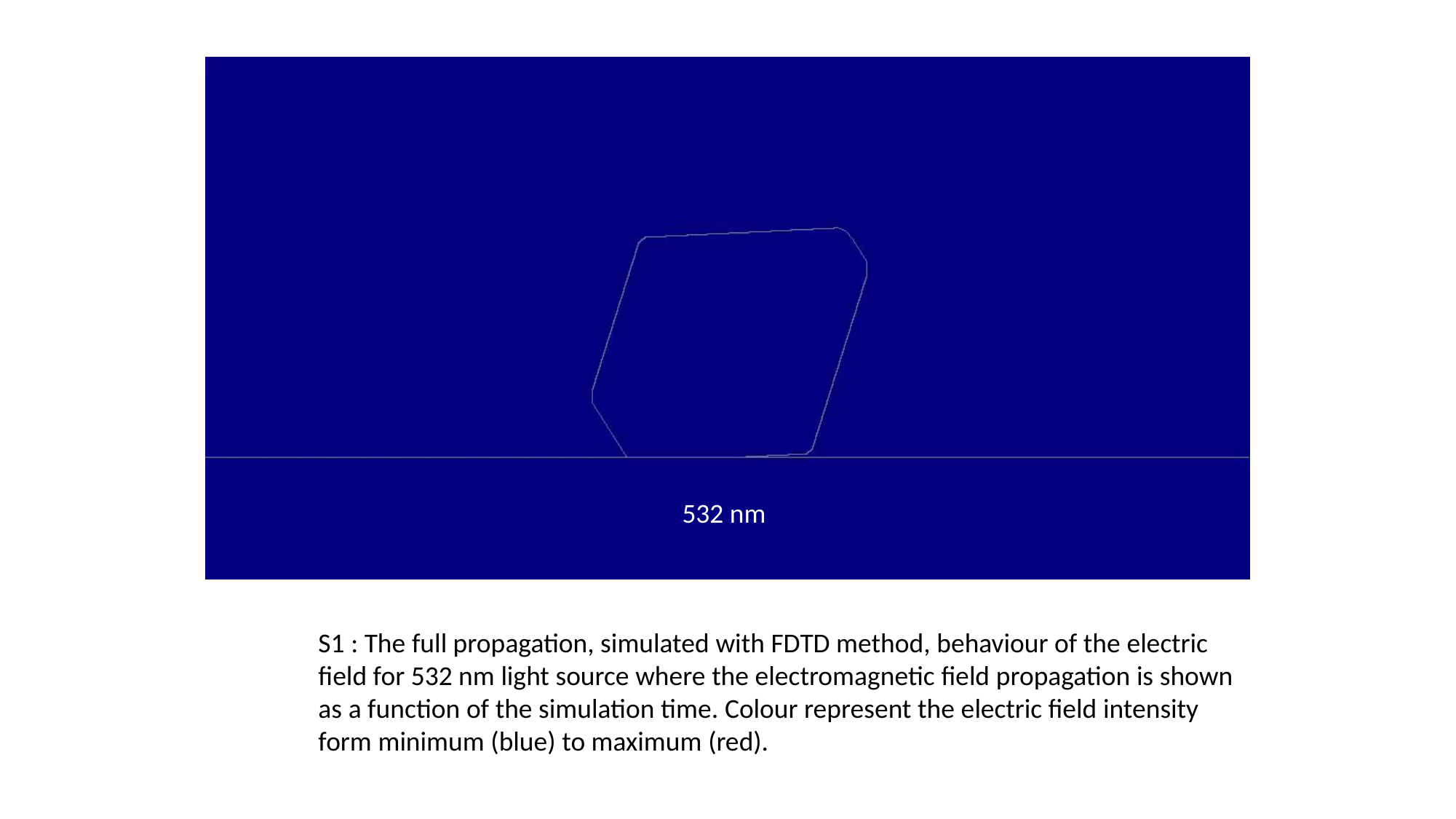

532 nm
S1 : The full propagation, simulated with FDTD method, behaviour of the electric field for 532 nm light source where the electromagnetic field propagation is shown as a function of the simulation time. Colour represent the electric field intensity form minimum (blue) to maximum (red).

## Slide 3
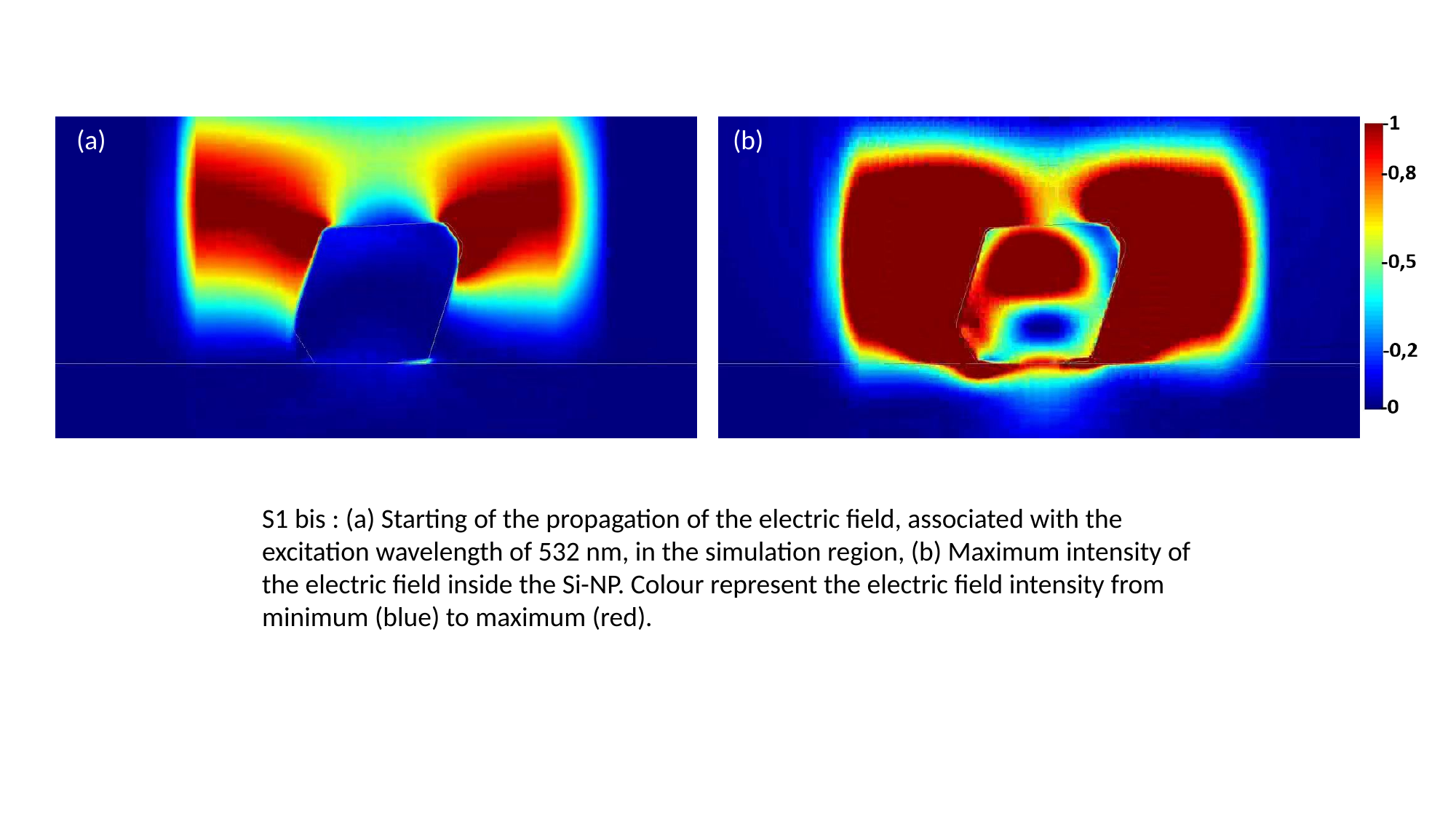

(a)
(b)
S1 bis : (a) Starting of the propagation of the electric field, associated with the excitation wavelength of 532 nm, in the simulation region, (b) Maximum intensity of the electric field inside the Si-NP. Colour represent the electric field intensity from minimum (blue) to maximum (red).

## Slide 4
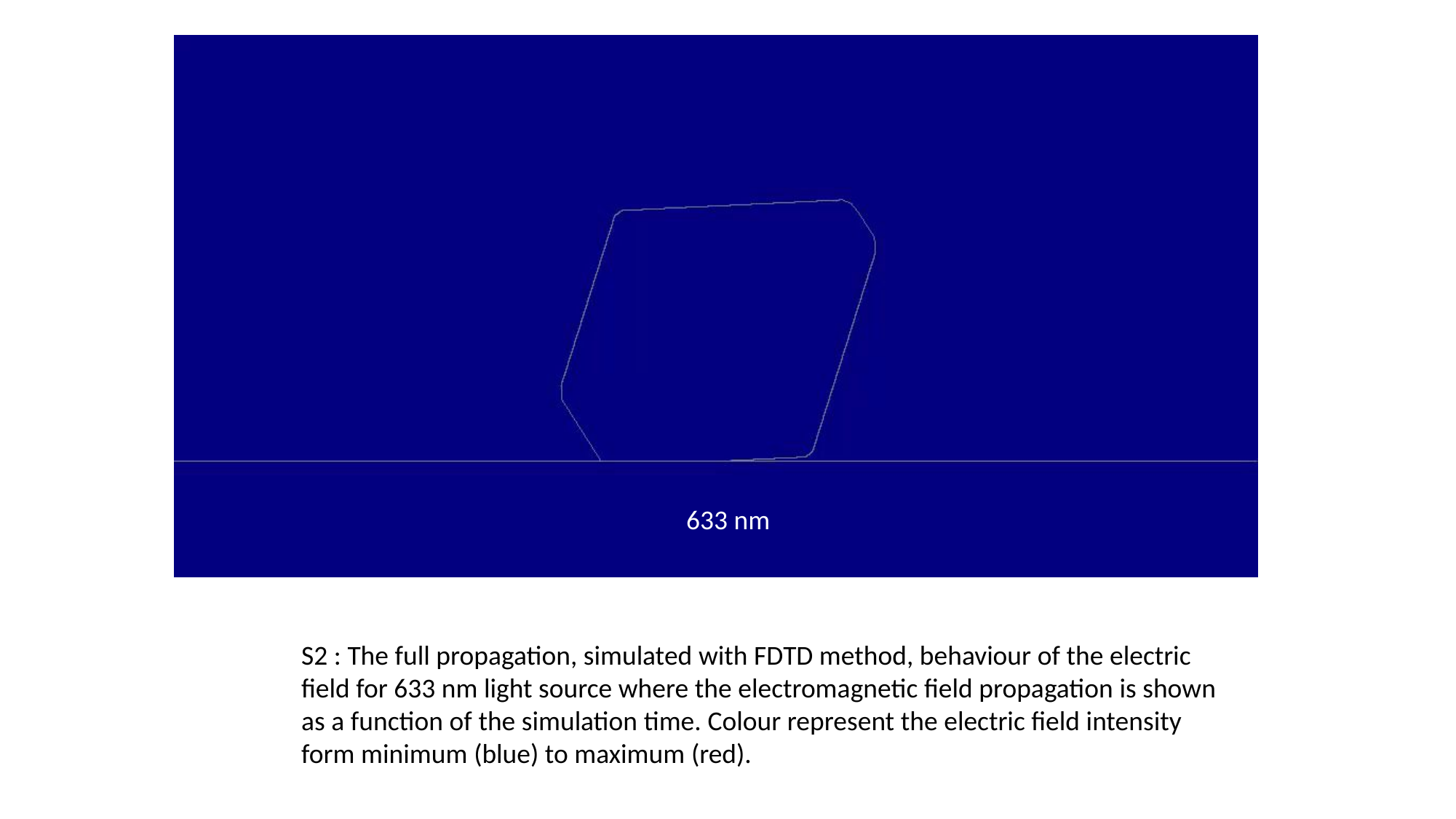

633 nm
S2 : The full propagation, simulated with FDTD method, behaviour of the electric field for 633 nm light source where the electromagnetic field propagation is shown as a function of the simulation time. Colour represent the electric field intensity form minimum (blue) to maximum (red).

## Slide 5
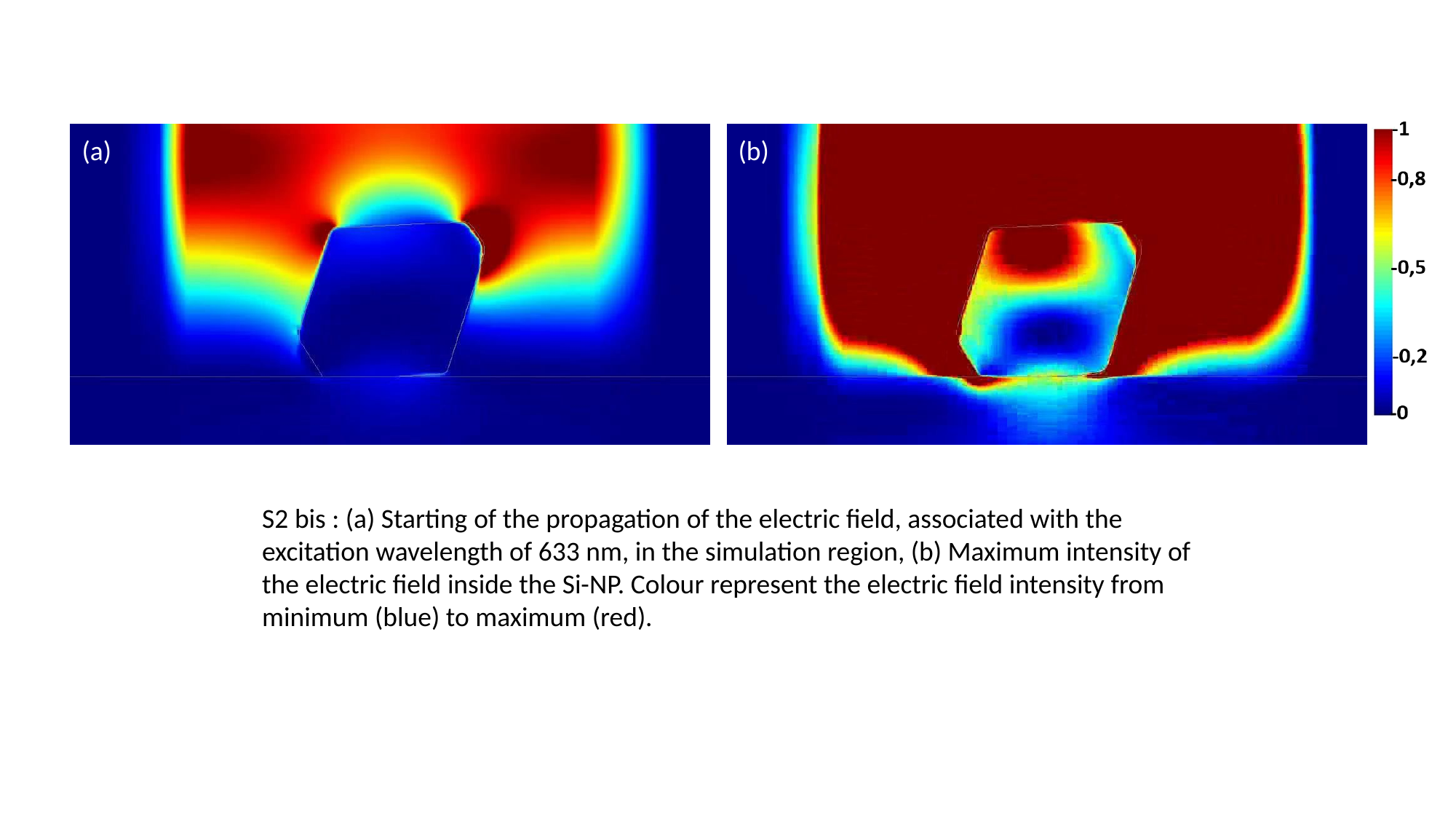

(a)
(b)
S2 bis : (a) Starting of the propagation of the electric field, associated with the excitation wavelength of 633 nm, in the simulation region, (b) Maximum intensity of the electric field inside the Si-NP. Colour represent the electric field intensity from minimum (blue) to maximum (red).

## Slide 6
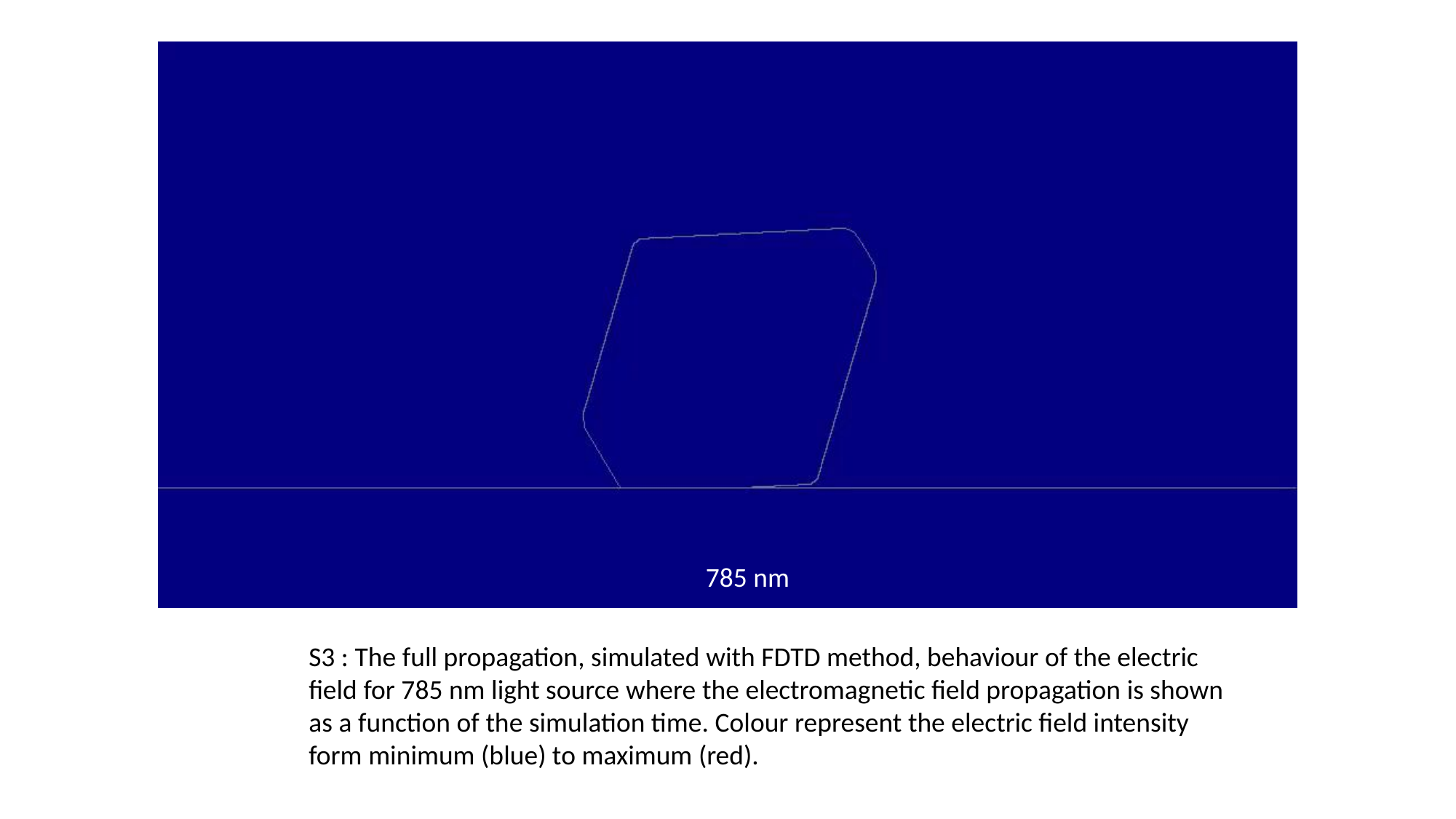

785 nm
S3 : The full propagation, simulated with FDTD method, behaviour of the electric field for 785 nm light source where the electromagnetic field propagation is shown as a function of the simulation time. Colour represent the electric field intensity form minimum (blue) to maximum (red).

## Slide 7
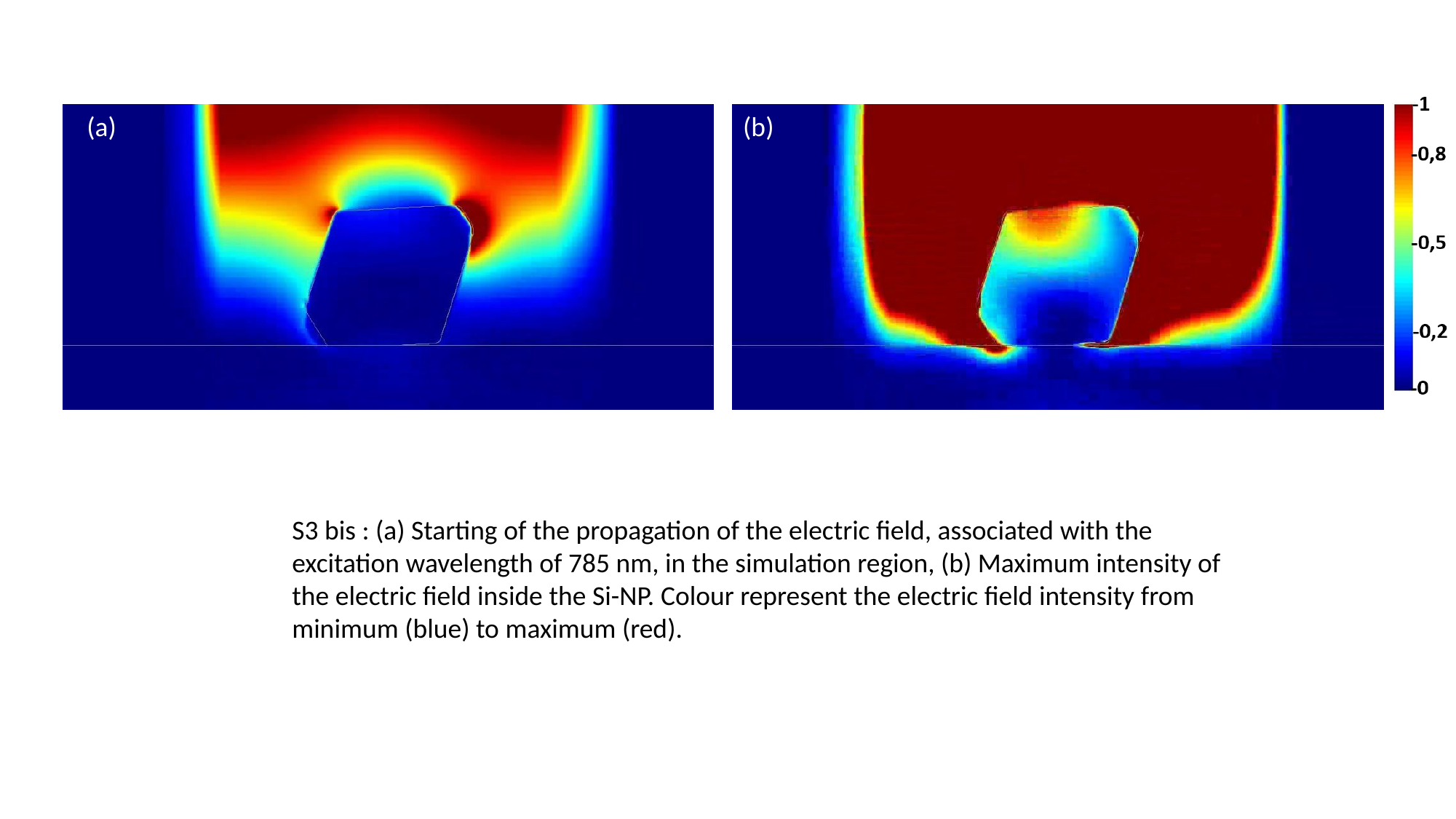

(a)
(b)
S3 bis : (a) Starting of the propagation of the electric field, associated with the excitation wavelength of 785 nm, in the simulation region, (b) Maximum intensity of the electric field inside the Si-NP. Colour represent the electric field intensity from minimum (blue) to maximum (red).
